# Progress in Laser Ablation and Biological Synthesis Processes: “Top-Down” and “Bottom-Up” Approaches for the Green Synthesis of Au/Ag Nanoparticles

**DOI:** 10.3390/ijms232314658

**Published:** 2022-11-24

**Authors:** Zhiwen Jiang, Liwei Li, Hao Huang, Wenbin He, Wuyi Ming

**Affiliations:** 1 Henan Key Lab of Intelligent Manufacturing of Mechanical Equipment, Mechanical and Electrical Engineering Institute, Zhengzhou University of Light Industry, Zhengzhou 450002, China; 2 School of Mechanical Science & Engineering, Huazhong University of Science and Technology, Wuhan 430074, China; 3 Department of Materials Science and Engineering, University of Virginia, Charlottesville, VA 22904-4745, USA

**Keywords:** laser ablation, green synthesis, Au/Ag nanoparticles, molecular structure, potential application

## Abstract

Because of their small size and large specific surface area, nanoparticles (NPs) have special properties that are different from bulk materials. In particular, Au/Ag NPs have been intensively studied for a long time, especially for biomedical applications. Thereafter, they played a significant role in the fields of biology, medical testing, optical imaging, energy and catalysis, MRI contrast agents, tumor diagnosis and treatment, environmental protection, and so on. When synthesizing Au/Ag NPs, the laser ablation and biosynthesis methods are very promising green processes. Therefore, this review focuses on the progress in the laser ablation and biological synthesis processes for Au/Ag NP generation, especially in their fabrication fundamentals and potential applications. First, the fundamentals of the laser ablation method are critically reviewed, including the laser ablation mechanism for Au/Ag NPs and the controlling of their size and shape during fabrication using laser ablation. Second, the fundamentals of the biological method are comprehensively discussed, involving the synthesis principle and the process of controlling the size and shape and preparing Au/Ag NPs using biological methods. Third, the applications in biology, tumor diagnosis and treatment, and other fields are reviewed to demonstrate the potential value of Au/Ag NPs. Finally, a discussion surrounding three aspects (similarity, individuality, and complementarity) of the two green synthesis processes is presented, and the necessary outlook, including the current limitations and challenges, is suggested, which provides a reference for the low-cost and sustainable production of Au/Ag NPs in the future.

## 1. Introduction

Advancements in nanotechnology in practically every field of study have simplified modern life. Structures, gadgets, and systems are all part of the increasing field of study known as nanotechnology [1,2]. Nanotechnology is a science and technology that uses a single atom or molecule to make substances [3,4,5]. After the invention of the scanning tunneling microscope in 1981, researchers used it to visually observe atoms and molecules [3], which has aroused great interest in the field of nanotechnology in the field of science. Therefore, nanotechnology is actually a technology that uses a single atom or molecule to make substances, representing a unique and innovative research method [6]. Its goal is to construct products with specific functions directly from atoms or molecules [7]. In general, nanomaterials can be classified into 0-D (e.g., core–shell nanoparticles), 1-D (e.g., coaxial nanowires), 2-D (e.g., graphene-based composites), and 3-D (e.g., mesoporous carbon-based composites), depending on their structure, as well as the outer shape of the material [8]. This means that the different heterogeneous nanostructured materials are based on structural complexity. Typically, spherically-shaped nanoparticles with diameters ranging from a few to tens of nanometers are referred to as 0-D nanostructures [8]. Nanoparticles (NPs) are an important area of nanotechnology and are materials with nanometer dimensions of less than 100 nm, which are considered 0-D-sized materials. NPs belong to the category of colloidal particle sizes, and they differ from macroscopic objects in that they have a large proportion of their size as surface area, and the surface atoms have neither long-order nor short-order amorphous layers. It can be considered that the atomic state on the surface of the nanoparticle is closer to the gaseous state [9].

**Figure 1:**
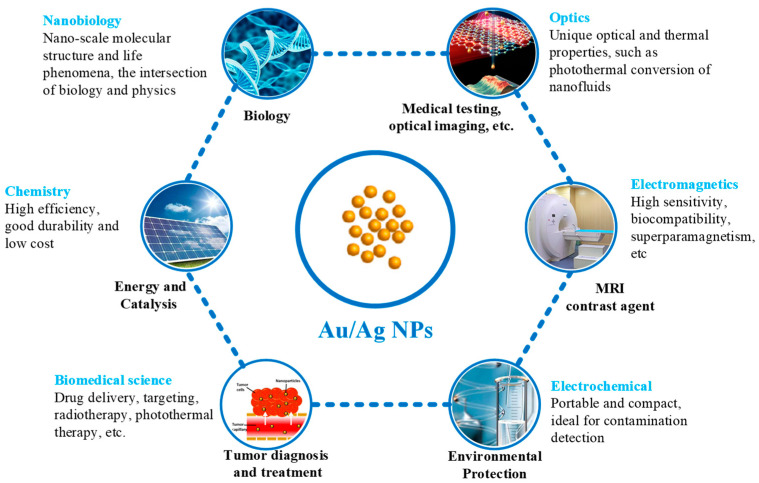
Properties and application of Au/Ag NPs in different fields.

Therefore, in the past decade, the rapid development of nanotechnology has attracted extensive attention in almost all industrial, agricultural, medical, and other fields. Among the various types of nanomaterials, metal nanoparticles (MeNPs) are particularly interesting materials because of their diverse properties for use in disease diagnosis and therapy [10], composite fabrication [11], catalysis [12], and sensor technology [13]. For example, gold nanoparticles (Au NPs) and silver nanoparticles (Ag NPs) are widely used in the medical field [14,15,16]. Au NPs are a kind of common metal nanoparticle, the size of which is usually 10–100 nm and occurs in different shapes, including gold nanospheres (Au NS), gold nanorods (Au NR), gold nanometer triangles, gold nanowires, nanogold shells, etc. To date, Au NPs are the most studied nanomaterials for biomedical applications. Similarly, the diameter of Ag NPs is in the range of 1–100 nm, and most of them are around 25 nm. Because they are extremely small particles and have a large surface area, it has significant surface effects, quantum-size effects, and quantum-tunneling effects, and their activity and permeability are also very strong. It exhibits antibacterial properties in infection treatment, wound healing, or drug delivery systems, with hundreds of times the bactericidal effect of ordinary silver [16,17,18]. In addition, due to the surface electronic properties of nanoscale metallic silver, it can form ligands with electron-withdrawing groups, such as sulfhydryl and amino groups, on bacterial protein molecules, thereby further enhancing the antibacterial effect. Figure 1 depicts the properties and applications of Au/Ag NPs in different fields, such as biology, medical testing, optical imaging, energy and catalysis, MRI contrast agents, tumor diagnosis and treatment, environmental protection, etc. In these fields, Au/Ag NPs are correspondingly involved in nanobiology, optics, chemistry, electromagnetics, biomedical science, electrochemistry, etc. For example, various optically-limiting nanomaterials, such as the Ag-Au alloy, have been produced by changing the material compositions [19]. Also, Au NPs with adjustable core surfaces are generated, which can be used to detect mercury ions selectively and sensitively in real systems [20]. In biomedical science, the Jiangsu Provincial Center for Disease Control and Prevention in China prepared a colloidal gold-based antibody detection kit using Au NPs with a diameter of 30 nm. The characteristics of the kit are simplicity, speed, and convenience, and the test results can be obtained in about 15 min [21].

**Figure 2:**
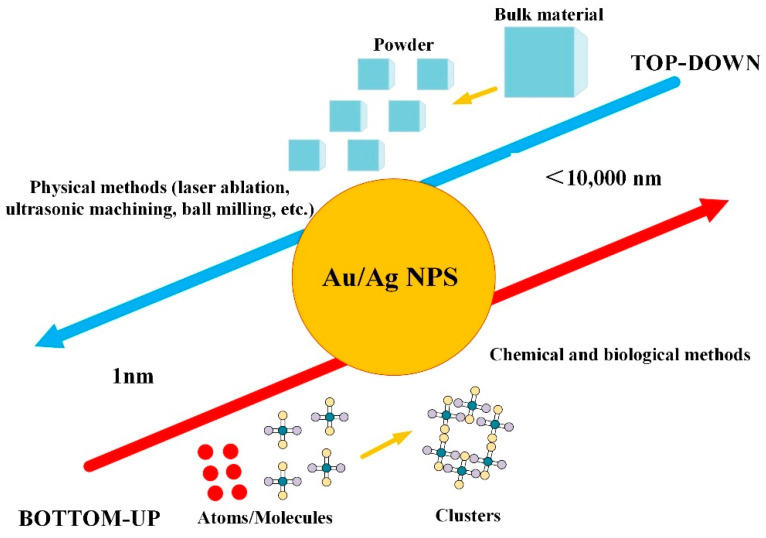
Two different methods to fabricate Au/Ag NPs: top-down and bottom-up.

In general, to achieve specific functions, Au/Ag NPs need to be composed of complex molecules with three-layer structures. For specific goals, the outer layer facilitates functionalization with other molecules like metal ions or polymers. The intermediate layer, which is chemically separate from the core layer, guarantees that the particle’s core and outer layers are bonded together. The nanoparticle’s core layer contains the nanoparticle’s substance, and its synthesis or processing method is the most important in its three-layer structure [22]. In general, Au/Ag nanoparticles can be synthesized by two different approaches: “top-down” and “bottom-up”, as shown in Figure 2. The two approaches are based on different techniques, depending on the materials and precursors applied. The “top-down” approaches based on converting bulk materials into small particles, such as physical methods (laser ablation, ultrasonic machining, ball milling, etc.), have the advantage of producing large quantities of nanoparticles, but this requires a lot of energy and expensive equipment investments. Whereas the “bottom-up” approaches involve the production of nanoparticles using chemical agents that assemble atoms into “seed” nuclei that will further grow into nanoscale clusters and particles, such as chemical and biological methods. The chemical method is characterized by low cost, simple operation, and strong scalability. Still, the chemical manufacture of stable and “pure” NP dispersions for biomedical or catalytic applications always necessitates extra purification steps to remove excess surfactant and leftover reactants before processing the functional nanomaterials [23]. Biological methods have the characteristics of high stability and biocompatibility, but the size, shape, and crystallinity of the nanoparticles synthesized by them cannot be well controlled.

Since the laser ablation process does not require molecular precursors, the yield is relatively high, and reagent costs are low. Usually, the price of 1 g of precursor gold chloride is about EUR 200, and the price of 1 g of gold foil is about half of the former [23]. Therefore, the laser ablation process is cost-effective after reaching a certain scale of mass production. This is very suitable for testing reagents produced in very large quantities, such as for the screening of new coronaviruses, which require lower costs. In addition, the Au/Ag NPs synthesized by biological methods have good biocompatibility and are very suitable for applications in the field of biomedicine. Therefore, the laser ablation and biotechnology synthesis of NPs represent two promising and sustainable green methods for the future. Hence, this study focuses on reviewing the principles of size and shape control when making materials under different methods of Au/Ag NP synthesis via laser ablation and biological methods. In addition, the applications of Au/Ag NPs in related fields, especially the biomedical field, are also reviewed. Finally, we compare the advantages and disadvantages of the two approaches: laser ablation and biological, and their scope of application, and make necessary projections for future technological development.

## 2. Fundamentals of the Laser Ablation Method

### 2.1. Brief Introduction

In 1987, Pailt et al. [24] reported the pioneering work on solid polylactic acid for material processing in closed liquids. In this study, oxide was used as a high-power pulsed laser-induced liquid-solid interface reaction quencher to create metastable materials for the first time. The process of the pulsed-laser-induced quenching of liquid-solid interface reactions and pulsed laser evaporation to form composite films were then covered in depth by Ogale et al. [25], which provided a theoretical framework for the creation of new techniques for the synthesis of nanostructures. The laser irradiation process in a liquid environment, also known as laser ablation in liquid (LAL), can greatly improve ablation efficiency [26]. The function of the liquids, such as water, involves heat transfer (conduction or convection) and bubble movement, which aids in the removal of the ablation particles that have been redeposited on the surface of the material and inhibits the fragmentation of chip oxidation. In addition, the liquid environment effectively cools the targets, preventing excessive heat buildup. Therefore, LAL can be widely used in laser cleaning and laser shock peening [27]. In recent years, the use of a liquid environment to control the surface quality and ablation efficiency of precious metals, such as gold and silver, has been a focus of laser processing. Therefore, this section focuses on the principle and mechanism of liquid-laser ablation, as well as the influence of size and shape control over NPs.

### 2.2. Laser Ablation Mechanism

The laser ablation of solid targets in a liquid environment allows for the generation of NPs with a variety of valuable properties, such as high-purity, easily functionalized surfaces, metastable compositions, or complex structures, including doped nanocrystals, core-shell, hollow microspheres, nano frills, or nano fluorescents [28]. At the same time, LAL has the following advantages: environmental sustainability, a simple experimental setup (no need for extreme conditions of a synthetic environment), and the lasting stability of nanoparticles, which are completely free from harmful pollutants or dangerous synthetic reactants. [29,30]. Au and Ag NPs were chosen as case studies because they are very important tools in nanotechnology, especially because of their optical and catalytic properties, the multiple possibilities of surface (biological) conjugation, chemical stability, and biocompatibility [31]. Furthermore, in recent years, there has been a lot of interest in the laser-assisted synthesis of Au and Ag NPs, and these nanoparticles can be used as reference materials for LAL.

**Figure 3:**
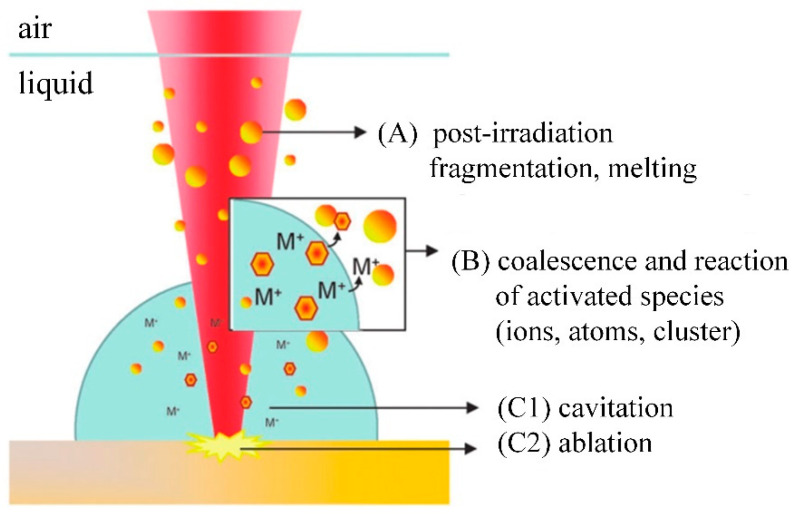
Mechanism of nanoparticle synthesis during the LAL process: (A) interaction of colloidal particles with laser beam; (B) polymerization and reaction of generated NPs with colloidal particles; (C1) generates an expansive cavitation bubble, within which crystallized NP is formed through nucleation and coalescence; (C2) incident laser pulse is adsorbed by the bulk material to form plasma. Reprinted/adapted with permission from Ref. [32]. Copyright 2014, Royal Society of Chemistry.

Many physicochemical reactions occur during the formation of NPs during the LAL process, which can be roughly divided into three categories [32]. These three types of reaction mechanisms are summarized in Figure 3. Mechanism A mainly postirradiates the colloidal mixture of the previously generated Ag-Au-NPs; mechanism B is the direct formation of Ag-Au-NPs during ablation; mechanism C is adsorbed by the bulk material through the incident laser pulse to form a plasma (C2), subsequently generating expanded cavitation bubbles, while crystalline NPs (C1) are formed inside the cavitation bubbles through nucleation and coalescence.

**Figure 4:**
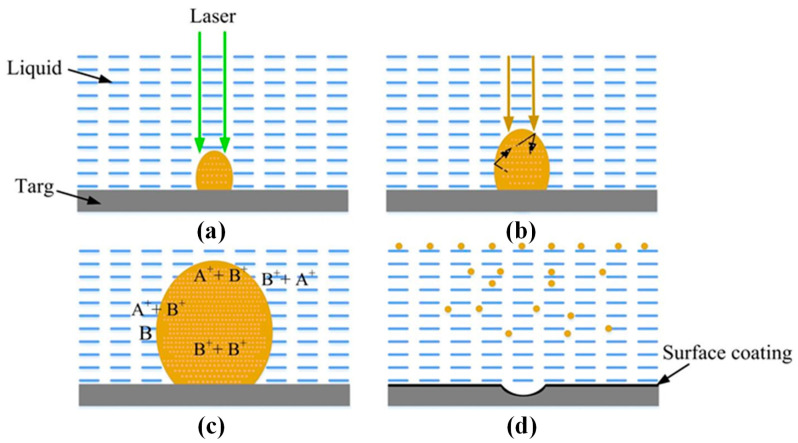
Evolution of laser-induced plasmas in liquids; (**a**,**b**) high-temperature chemical reaction between the target ablation; (**c**) the laser-induced plasma and the liquid; (**d**) chemical reaction inside the liquid. Reprinted/adapted with permission from Ref. [33]. Copyright 2007, Elsevier.

The most important difference between laser-ablating solids in a vacuum or dilution gas and liquid ablating is that liquid restricts the movement of the plasma plume. Therefore, a series of processes, such as the generation, transformation, and condensation of the plasma plumes generated by solid laser ablation in a liquid environment, have taken place under the constraint of liquid. Importantly, the constraints of the liquid will greatly affect the thermodynamic and dynamic properties of the evolution of the plasma plume, resulting in a different environment for the formation of the condensed phases of the laser-ablated solids in a vacuum or diluted gas [33]. Figure 4 shows the evolution of laser-induced plasma in liquid; Figure 4a shows the laser-induced plasma generated by the front of the laser pulse shining on the target; Figure 4b shows that the plasma plume expands in the liquid due to absorbing the plasma-induced pressure generated by the laser pulse and shock wave in the later period; Figure 4c shows the four chemical reactions occurring in the plasma and liquid, as well as the interface between the plasma and liquid; Figure 4d shows two kinds of condensation of a plasma plume in liquid: one is used to prepare a surface coating on the target, and the other is used to prepare nanoparticles in the liquid. 

**Figure 5:**
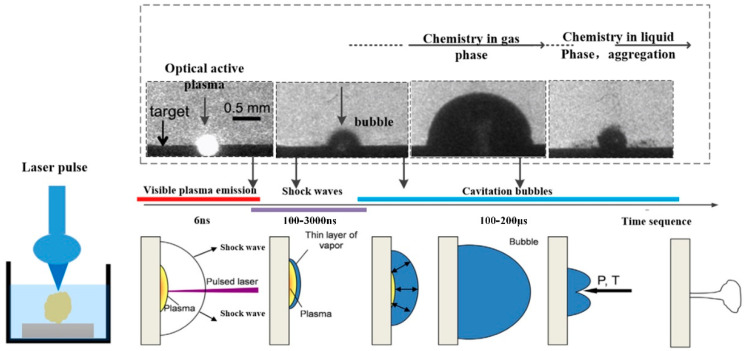
Time duration of plasma emission, shock wave formation, and bubble collapse during the LAL process. Reprinted/adapted with permission from Ref. [36]. Copyright 2015, Elsevier.

**Figure 6:**
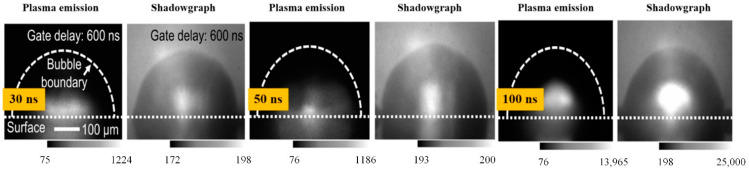
Plasma emission images after laser irradiation for 600 ns (pulse durations were 30 ns, 50 ns, and 100 ns, respectively). Reprinted/adapted with permission from Ref. [39]. Copyright 2015, AIP Publishing.

When the laser ablation of a solid target produces nanoparticles in the liquid, the temperature rise of the liquid will have a certain influence on the formation of cavitation bubbles and particles. Menendez et al. [34] analyzed the effect of temperature on the hydrodynamic diameter of nanoparticles. The effect of the physical properties of the liquid on the hydrodynamic diameter of the nanoparticles was investigated. Ageev et al. [35] found that laser-etched cavities at long pulses (1 ms) or high pulse rates (50 kHz) were wide and produced shallow bubbles and absorbed debris due to light scattering. Dell’Aglio et al. [36] discussed the mechanisms involved in LAL technology in detail based on some basic experiments. The time duration of each process in the LAL is depicted in Figure 5. After laser-matter interaction, laser-induced breakdown occurs first, followed by the formation of a shock wave [37], plasma expansion and cooling, and finally, cavitation bubble formation, expansion, and collapse [38]. When the bubble breaks, the nanoparticles generated in the plasma-cooling phase will diffuse into the surrounding liquid, forming a colloidal solution. The relationship between the primary plasma and the bubble was examined by Tamura et al. [39], and they discovered that the pulse duration had a significant impact on the amount of plasma quenching during the first stage. Figure 6 shows the plasma emission image, shadow image, and emission spectrum obtained after laser irradiation at 600 ns. The pulse duration used was 30, 50, or 100 nanoseconds. When the pulse duration was the longest, i.e., 100 ns, a narrow spectral line was obtained. It could be seen from the plasma emission image that the plasma emission intensity obtained by a 100 ns pulse was higher than that obtained by a 30 or 50 ns pulse. After 600 ns of pulse irradiation, the bubble size obtained by different pulse durations was basically the same. The emission was observed below the target surface in the image. This was the reflection of light on the target surface. It was difficult to observe the optical emission of the plasma in a direction that was completely parallel to the target surface.

### 2.3. Size and Shape Control

Research on laser-fabricated nanoparticles in liquid environments focuses on the characterization of nanoparticles, especially their size and shape. The size and shape of nanoparticles directly affect their properties (catalytic performance, surface activity, etc.). So far, many metal nanoparticles have formed reliable preparation methods, but understanding how to control the size and shape of nanoparticles will be a major focus and challenge in future research.

**Figure 7:**
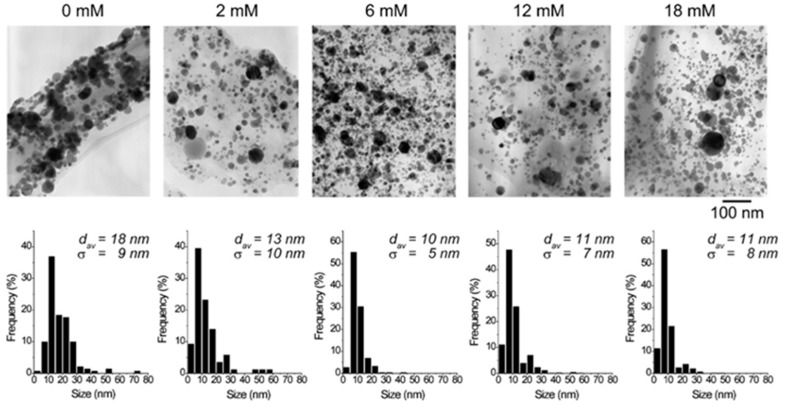
TEM images and the particle size distributions of silver nanoparticles prepared by laser ablation using a silver plate in PVP solutions at various concentrations. Reprinted/adapted with permission from Ref. [31]. Copyright 2008, Elsevier.

**Size control:** The existing research mainly focuses on the high reaction characteristics of nanoparticles to control their size. Henglein et al. [40] created gold NPs in early research into laser-based nanoparticle synthesis via the ablation of gold thin films at a wavelength of L = 694 nm using a pulsed ruby laser (sized between 5 and 15 nm). Mafune et al. [41] produced colloids of silver (10 nm particle size) and gold (8 nm particle size) using a Nd: YAG laser with a pulse duration of t = 10 ns, and a wave-length of L = 532 nm. By introducing chloride into the liquid environment during the laser ablation, Prochazka et al. [42] were able to avoid the production of larger colloidal particles and reduce the average particle size (11 nm, silver). Polyvinylpyrrolidone (PVP) can increase the formation efficiency and stability of Ag NPs, as shown by Tsuji et al. in their study [43]. The TEM images and particle size distribution of Ag NPs produced at various PVP concentrations are shown in Figure 7. Tomko et al. [44] revealed that the height of the liquid column and the ambient pressure have a greater impact on the maximum bubble radius and collapse time, thereby affecting the particle size distribution of nanoparticles. The size of nanoparticles prepared by laser in a liquid environment is mostly below 100 nm, which can achieve size control below 100 nm.

**Shape control:** Utilizing lasers to process nanomaterials allows for better shape control. Setoura et al. [45] used optical spectroscopy and scanning electron microscopy (SEM) to measure the morphological changes in single Au nanoparticles under the excitation of a focused 488 nm continuous laser. The heating rate under continuous laser irradiation is much lower than that under pulsed laser irradiation, which allows for better control over the heating and morphology changes of nanoparticles. In summary, laser nanoparticle preparation in a liquid environment can provide good size and shape control. Table 1 summarizes the shape and size of Au/Ag NPs in the typical literature in recent years.

### 2.4. Summary

This section mainly reviews the main preparation methods (LAL) of gold and silver nanoparticles, preparation principles and mechanisms, and the influence of shape and size control over nanoparticles. Size control of noble metal nanoparticles prepared by laser ablation can be achieved by adding specific molecules to the preparation environment in water that physically or chemically interact with the particle-forming surface to limit the growth of nanoparticles (Ag, Au). It was found that the particle size of noble metals (Au, Ag) was successfully limited by using ionic surfactants [18], sodium chloride [16], etc. However, the specific mechanism that limits particle growth requires further study. In conclusion, LAL-prepared nanoparticles (gold, silver, etc.) are well characterized but still lack size control and stability, especially in pure water. In addition, the physical and chemical properties of the surface of NPs are closely related to their application scenarios, which require in-depth research.

## 3. Fundamentals of Biological Method

### 3.1. Brief Introduction

In 1999, Gardea-Torresdey [48] used the plant reduction method (alfalfa extract) for the first time to prepare face-centered cubic tetrahedron, hexagonal sheet, icosahedral polytwins, decahedron polytwins, and irregularly shaped Au NPs. The biosynthesis of precious metal nanomaterials, such as gold and silver, has become a hot spot in the field of nanotechnology in recent years. Moreover, because the reducing agents are based on plants, bacteria, fungi, yeast, or microorganisms, biosynthetic Au/Ag NP production appears to have a lower environmental effect; therefore, it is a green synthesis method that has now developed into an important synthetic strategy. Furthermore, natural chemicals derived from plant roots or microbes are also employed to decrease metal salts used as precursors. Compared with the physical method, the biosynthesis of raw materials is simple, economical, environmentally friendly, and easy to scale up to achieve high yields. In this section on biological methods, the review will mainly cover plant-mediated synthesis, microbe-mediated synthesis, and algae-mediated synthesis.

### 3.2. Synthesis Principle

**Figure 8:**
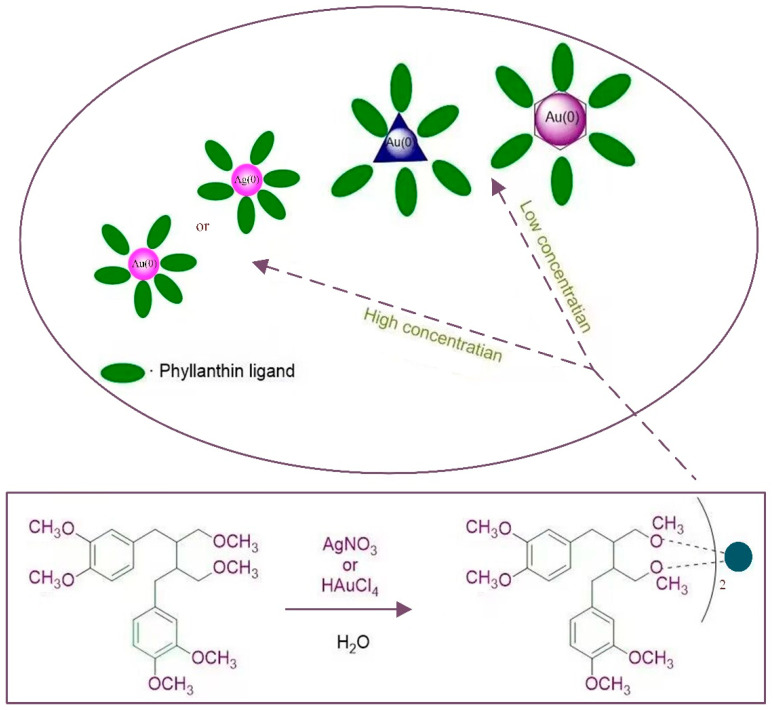
Schematic diagram of the formation of phyllanthin stabilized Au/Ag NPs.

**Plant-mediated synthesis:** Metal salts employed as precursors in the fabrication of Au/Ag nanoparticles were reduced by plants harboring certain bioactive chemicals. Flavonoids, ketones, amino acids, phenols, aldehydes, and vitamins, which are contained in the chemical structure of plants, have the ability to reduce precious metal ions. Kasthuria et al. [49] prepared anisotropic gold and spherical/quasispherical silver nanoparticles (NPs) by reducing chloroauric acid (HAuCl_4_) and silver nitrate (AgNO_3_) aqueous solutions with phylloxacin extract, as shown in Figure 8. They found that the size and shape of the NPs could be controlled by varying the concentration of the phylloxacin extract, resulting in the formation of hexagonal or triangular gold nanoparticles with a diameter distribution ranging from 20 to 100 nm (mostly around 30 nm). The experimental results showed that the shape of the gold nanoparticles changed from hexagonal to spherical with the increase in the initial concentration of phylloxacin extract. Fourier transform infrared spectroscopy and thermogravimetric analysis indicated that there was an interaction between the nanoparticles and the phylloxacin extract. Chen et al. [46] analyzed the stability of the prepared NPs and found that their structure was easily destroyed by oscillation, resulting in the slow growth and formation of one-dimensional Ag nanochains. Basavegowda et al. [50] observed a rapid reduction in chloroauric acid ions when treating an aqueous solution of chloroauric acid with pomegranate fruit extract as a reducing agent, resulting in the formation of highly stable nanoparticles in the solution with sizes ranging from 10 to 50 nm. Experiments in UV-vis spectroscopy showed that these nanoparticles showed an absorption peak at 536 nm, corresponding to the plasmon resonance of Au NPs. In addition, in vitro experiments showed that the synthesized Au NPs were active against Streptococcus and Escherichia coli. Zhan et al. [51] prepared Au NPs via a biological method using Cacumen platycladi leaf extract. The results showed that flavonoids and reducing sugars were the main reducing and protecting components in the extract and were crucial for the biosynthesis of Au NPs. Furthermore, the pH of the reaction solution proved to be the most important factor affecting the synthesis process. Some researchers have found that coriander [52] and spinach [53] can also be used to prepare Au NPs. Pahal et al. [54] prepared Au NPs and gold-silver bimetallic nanoparticles (Au-Ag BMNPs) from the water leaf extract of wheat. The analysis showed that these two kinds of NPs were highly crystalline and ranged from spherical to elliptical. The size of Au NPs was in the range of 5–40 nm, while that of Au-Ag BMNPs was in the range of 5–30 nm. Most of the synthesized Au NPs are less than 100 nm and have good stability. However, their large-scale mass production and the economics of their manufacturing need to be further studied. Shankar et al. [55] exposed sterile geranium leaves to water ions from chloroauric acid and observed a rapid reduction in metal ions, resulting in the formation of stable Au NPs of variable sizes (20~40 nm) that took on a variety of shapes, including rods, flat sheets, and a triangular shape. However, the exact reason for its shape change is unknown.

**Figure 9:**
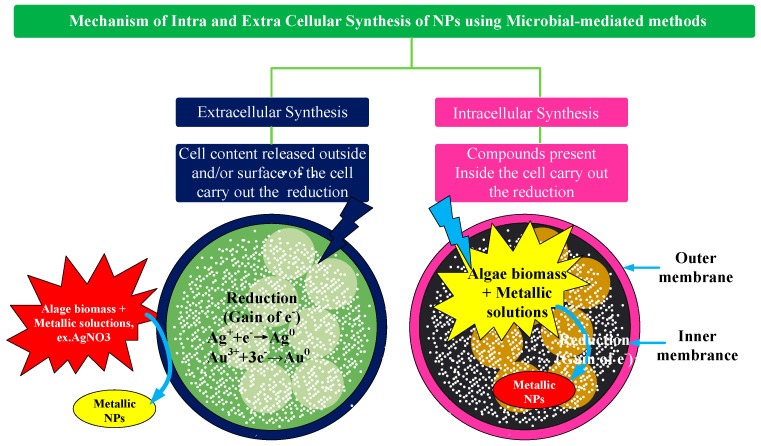
Mechanism of intracellular and extracellular microbes mediated synthesis of NP. Reprinted/adapted with permission from Ref. [55]. Copyright 2016, Elsevier.

**Microbes-mediated synthesis:** Microbes biosynthesize NPs by grabbing target ions from their surroundings, which are subsequently transformed into elemental metal atoms by enzymes produced by cellular activity. Depending on the site of NP creation, microbial-mediated NP synthesis occurs intracellularly or extracellularly, and the synthesis method is depicted in Figure 9. Metal ions are captured on the cell surface and reduced in the presence of enzymes in extracellular NP production, whereas metal ions are carried inside the microbial cells in the presence of enzymes to create NPs in intracellular NP synthesis [55]. Gerick and Pinches [56] demonstrated the intracellular synthesis of gold nanoparticles of different morphologies and sizes in two fungal cultures (V. luteoalbum and isolate 6–3). They found that by controlling parameters, such as pH, temperature, gold concentration, and AuCl_4_ exposure time, the rate of particle formation, as well as the size of the nanoparticles, could be controlled to some extent. Also, the extracellular biosynthesis of Ag NPs using the fungi Aspergillus fumigatus and Fusarium hemiarchitecture has been reported by some researchers [57,58]. Basavaraja et al. [57] reported that the extracellular synthesis of Ag NPs (i.e., by reduction of Ag^+^ to Ag) from a silver nitrate solution using the fungus “Fusarium hemitectum” produced highly stable and crystalline Ag NPs. Transmission electron microscopy revealed that they were between 10 and 60 nm in size and were mostly spherical. Interestingly, the colloidal suspension of Ag NPs was stable within a few weeks. Mukherjee et al. [59] revealed the formation mechanism of NPs synthesized by fungal enzymes, in which species-specific NADH-dependent reductase was released by Fusarium oxysporum. The experimental results showed that the proposed method could be successfully applied to the extracellular reduction of gold ions to GNP, and the size distribution of Au NPs was between 8~40 nm. The greatest advantage of this method based on fungal enzymes is that it is possible to develop a reasonable method for the synthesis of NPs from a series of components, such as oxides or nitrides. Actinomycetes are microorganisms with important characteristics of fungi and prokaryotes, although they are classified as prokaryotes. Ahmed et al. [60] investigated the formation process of a novel alkali-resistant actinomycete for the rapid intracellular synthesis of monodisperse Au NPs. Experimental results demonstrated that the formation of the NPs was affected by extreme biological conditions, such as alkalinity and a slightly elevated temperature [60,61]. In general, different microorganisms have different NP formation mechanisms, grabbing target ions from the environment on the microbial cell surface or inside of it and then reducing the metal ions to NPs in the presence of enzymes produced by cell activity [62].

**Figure 10:**
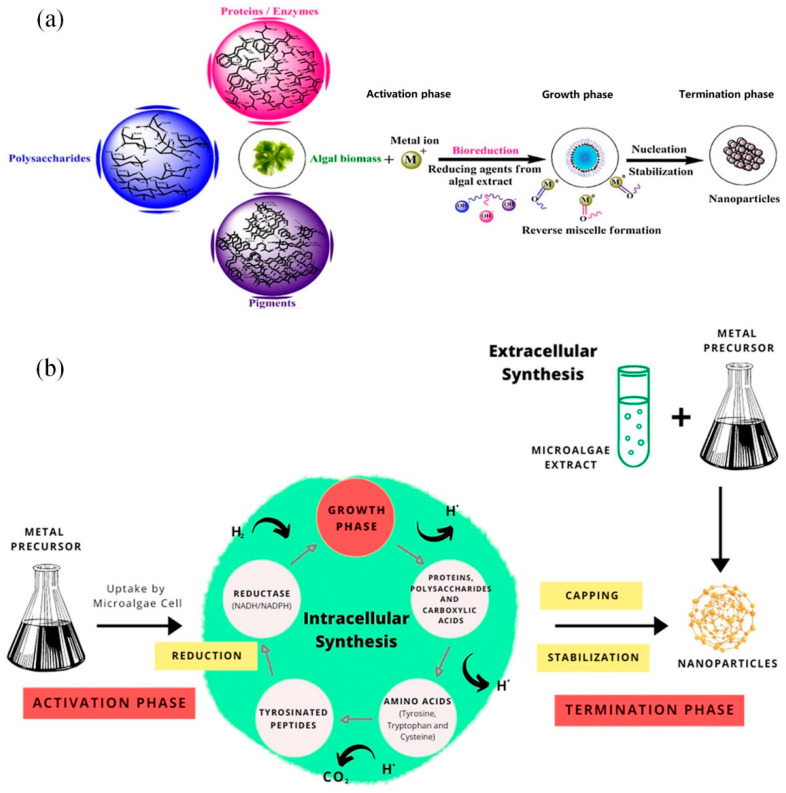
(**a**) General mechanism of the algae-mediated biosynthesis of metal nanoparticles Reprinted/adapted with permission from Ref. [55]. Copyright 2016, Elsevier. (**b**) Schematic diagram of the intracellular and extracellular synthesis mechanisms of algae-mediated nanoparticles. Reprinted/adapted with permission from Ref. [68]. Copyright 2020, Elsevier.

**Algae mediated synthesis:** Marine algae is rich in lipids, minerals, and certain vitamins, as well as a variety of biologically-active substances such as proteins, polysaccharides, and polysaccharides, and is a well-known functional food. They have potential medicinal value against thrombosis, allergy, cancer, lipidemia, oxidative stress, hypertension, and other degenerative diseases [63,64,65]. Diatoms and sponges, one of the biological entities found in marine resources, are typical nanostructures, which are composed of silica and the nanostructured covering of coral reefs. Thus, algae are also used as “biofactories” for the synthesis of Au/Ag NPs. Among the different types of bioreductants, algae have obvious advantages due to their macroscopic structure, high metal uptake capacity, and low cost [66]. The general mechanism and overview of the metal nanoparticle biosynthesis mediated by algae are shown in Figure 10a [55]. Polysaccharides, proteins/enzymes, and fragments in algae can be used as catalysts, reducing agents, and capping agents for metal ions [67]. The common activation, growth, and termination stages of metal ions are reduced through biological processes, as shown in Figure 10b [68]. The reduction of metal ions, like Ag+ or Au+, is followed by the nucleation of the reduction of Ag or Au atoms during the activation phase. The growth phase is characterized by the spontaneous coalescence of small neighboring NPs into larger particles as well as the maintenance of thermodynamic stability, and the termination phase consists of the final shape of NPs [55].

**Figure 11:**
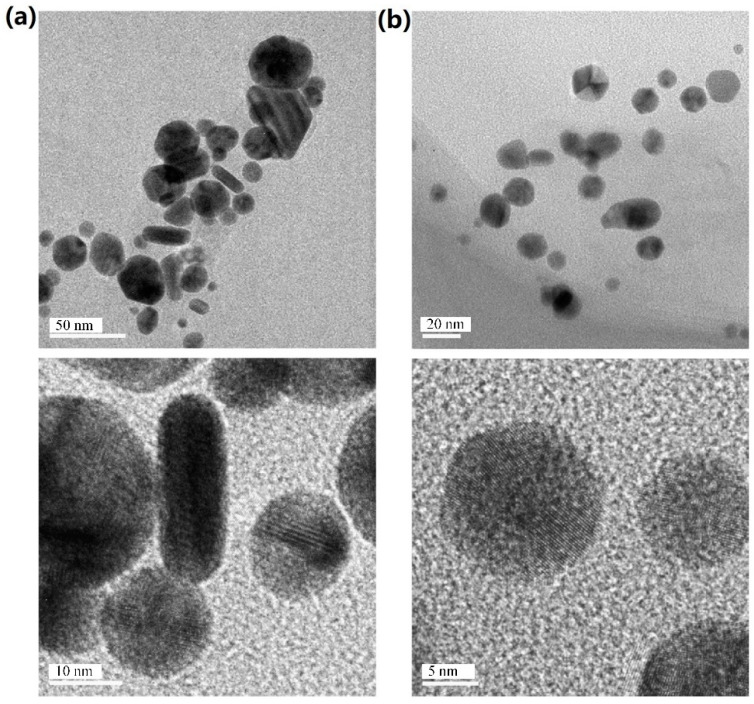
TEM micrographs of Au NPs produced by *F. vesiculosus* in pH 4 (**a**) and pH 7 (**b**) solutions. Reprinted/adapted with permission from Ref. [70]. Copyright 2009, Elsevier.

Venkatesan et al. [69] reported that Au NPs with biomolecular functionalization of primary amino groups, hydroxyl groups, and other stable, functional groups were rapidly synthesized (within 1 min) by reducing chloroauric acid with a novel Ecklonia cava. The X-ray diffraction pattern confirmed that the Au NPs had high purity and a face-centered cubic structure, and microscopic observation showed that they had an average particle size of 30 ± 0.25 nm and were spherical and triangular in shape. The synthesized gold nanoparticles have good antibacterial and good biocompatibility with human keratinocyte cell lines. Mata et al. [70] investigated the reduction of Au (III) to Au (0) using the brown alga Fucus vesiculosus. They found that metallic gold existed in the form of microprecipitates on the biomass surface and in the form of nanoparticles in solution. Additionally, the experimental results showed that the shape of the nanoparticles could be regulated by adjusting the pH value. For example, the uniform spherical nanoparticles produced at pH 7 were better than those obtained at pH 4, shown in Figure 11. Prasad et al. [71] confirmed the synthesis of Ag NPs using the Australian brown alga Nostoc algae. Extracts of this algae were used as reducing and stabilizing agents. The size of the synthesized Ag NPs ranged from 75 to 2000 nm, and the X-ray diffraction pattern showed that the formed Ag NPs had a face-centered cubic structure.

### 3.3. Size and Shape Control

Another essential aspect of nanoparticles is their size, which is linked to their form and surface [72]. The particle’s relatively small size permits them to pass through the circulatory system and, if internalized, reach the damage site. The tiny size of the particles ensures increased permeability through the skin in the event of external devices, such as burns. Nanoparticles should be smaller than or equal to 100 nm to reach the target tissue. The influence of size, as indicated in the example, is another factor to consider. The antibacterial action of Ag NPs has been demonstrated to increase as their size decreases. Nanoparticle shape is the most important attribute that affects the surface-to-volume ratio as well as the distribution of tiny particles in the body [16]. In the case of Ag NPs, the shape is also a property that can change the bacterial reaction, which can be utilized for drug delivery applications, tissue regeneration, photocatalytic applications, and cancer treatment [73].

**Figure 12:**
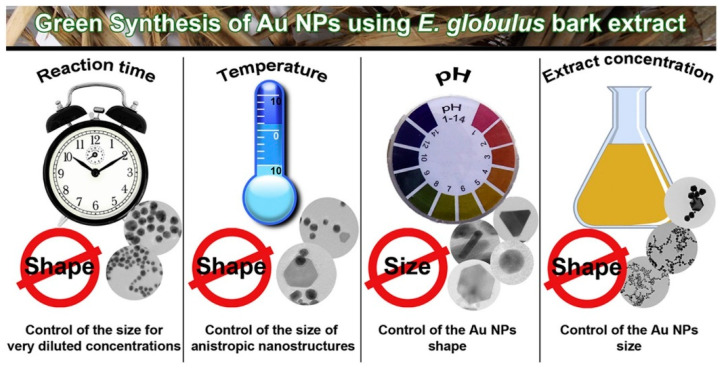
Effects of reaction time, temperature, pH, and extract concentration on the biosynthesis of Au NPs. Reprinted/adapted with permission from Ref. [74]. Copyright 2017, Elsevier.

Kasthuri et al. [49] found that the size and shape of Au NPs could be controlled by varying the concentration of the phylloxacin extract. The extract containing HAuCl_4_ has a low concentration and a slow reduction rate, forming hexagonal, triangular Au NPs. The particle size distribution shows that the main peak is between 32 and 43 nm, the particle size range is 10–110 nm, and the average particle size is 37.5 nm, as shown in Figure 12. The shape of the Au NPs changed from hexagonal to spherical with an increase in the concentration of the phylloxacin extract. Pinto et al. [74] used Eucalyptus globulus Labill. bark aqueous extract to conduct a detailed study on the effects of experimental parameters (temperature, pH, extract concentration, and reaction time) on the biosynthesis of Au NPs in order to establish a reasonable meth-od to fine tune the properties of Au NPs obtained from the plant extracts, aiming to fully control the size and morphology of NPs. The results obtained show that the concentration of the extract plays a major role in the size of gold nanoparticles (for very low concentra-tions, it can reach a “size plateau”) and that the pH value of the medium has a slight im-pact on their morphology, as shown in Figure 12. Prasad et al. [71] observed a change in the size of Ag NPs with temperature. Agglomeration of nanoparticles was observed at high temperatures. The average size of Ag NPs formed at a certain temperature (<65 °C) was 75 nm, while at higher temperatures (greater than 95 °C) it was larger than 2 μm. Table 2 lists the comparison between size and shape in the biological synthesis of Au/Ag NPs. It can be seen from Table 2 that there are obvious differences in the shape and size of Au/Ag NPs through different biosynthesis methods, which also provides a feasible approach for us to control the size and shape of nanoparticles.

### 3.4. Summary

This section reviews three representative biosynthesis methods for creating Au/Ag NPs, as well as their mechanisms and the control of their shape and size. According to other reviews, the biological reduction process offers benefits such as ecofriendly environmental protection, simplicity and speed, no separation after preparation, and good nanomaterial stability. With human keratinocyte cell lines, the produced Au/Ag NPs demonstrated good antibacterial and biocompatibility. Because of this, Au/Ag NPs have a wide range of potential applications in the disciplines of drug delivery, tissue engineering, and biosensors, according to their physicochemical characteristics. An alternative ecologically acceptable method for recovering gold from diluted hydrometallurgical solutions and creating Au NPs of various sizes and shapes is bioreduction, employing bacteria.

## 4. Applications

Due to their unique properties, gold/silver nanoparticles have been widely used in reimaging, sensing, drug release [73,74], tumor diagnosis and treatment [76], and other fields. This section briefly reviews the typical applications of laser ablation processing and biosynthetic gold/silver nanoparticles in these fields.

### 4.1. Biology

**Figure 13:**
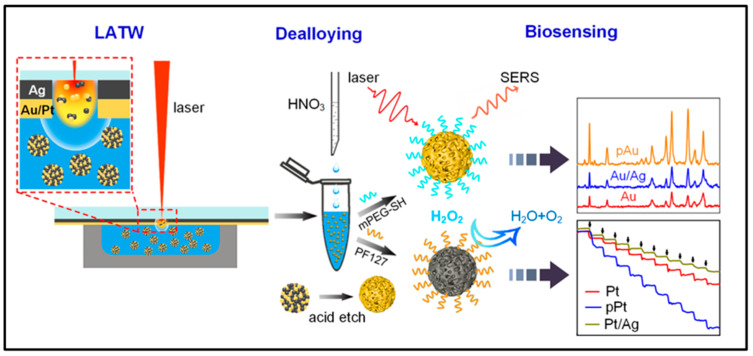
Schematic illustration of the fabrication process of pAu NPs and pPt NPs using LATW and dealloying for biosensing applications. Reprinted/adapted with permission from Ref. [79]. Copyright 2022, Elsevier.

NPs have been the focus of various research activities in nanomedicine, profiting from their tailor-made size, shape, surface chemistry, and physicochemical properties. They have emerged as promising candidates for the vectorization of biomolecules, for bioimaging, and as theranostic agents [77]. The safety, absorption, and biological behavior of human cell lines generated by laser-fabricated Au NPs in water or polymer solutions were assessed by Corrard et al. [78]. The study demonstrated that the laser ablation of Au NPs in human cell lines is safe and effective as a therapeutic measure. Yang et al. [79] proposed a synthetic strategy that combines laser ablation toward water (LATW) and the deprivation of porous precious metal NPs, as shown in Figure 13. This strategy can give the precious metal NPs a nanopore structure and has the potential to promote the further development of fields such as plasma, biomass sensing, optical therapy, and catalysis. The effectiveness of Ag NPs produced by pulsed laser ablation (PLA) in an aqueous medium against several Gram-positive and Gram-negative/Gram-positive human intestinal pathogenic bacterial strains was investigated by Pandey et al. [80]. The findings unequivocally demonstrated the production of Ag NPs by the antibacterial activity of PLAs and demonstrated that it could be employed as a potent growth inhibitor against a variety of harmful bacteria in a variety of medical devices and antibacterial control systems. Torrisi et al. [81] conducted the synthesis of Au NPs using an Nd: YAG pulsed laser, and certain physical characterization techniques were reviewed, along with some of the uses of Au NPs in radiation, biomaterials, the preparation of specific targets and optics. According to research by Haque et al. [82], biosynthetic precious metal nanoparticles, particularly those made of gold and silver, exhibit effective angiogenesis-promoting and restraining properties. These properties make them useful in the treatment of cancer, cardiovascular diseases, ischemic diseases, wound healing, and other conditions. In order to create gold nanoparticles in an environmentally friendly manner, Kumar et al. [83] investigated their catalytic reduction of 4-nitrophenol using an Andean bee pollen water extract. The findings of the experiments demonstrated that Au NPs could efficiently convert the organic pollutant 4-NP into 4-aminophenol.

### 4.2. Tumor Diagnosis and Treatment

**Figure 14:**
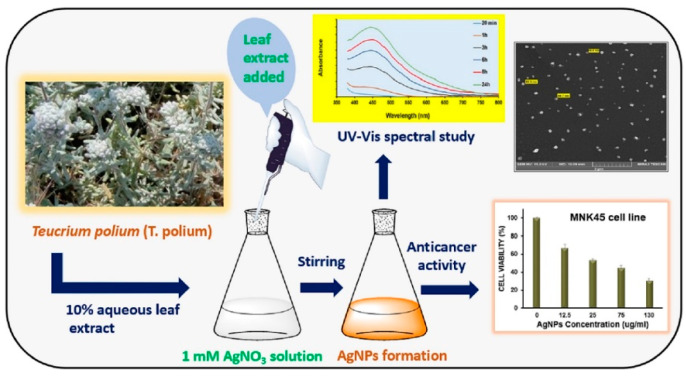
Leaf extract and synthesized nanoparticles of medicinal plant poliomyelitis and their anticancer activity. Reprinted/adapted with permission from Ref. [85]. Copyright 2020, Elsevier.

The transport of nanoparticles to areas where tissue disease is present allows a better view of the diseased tissue, and it can be used as targeted radiation or heat therapy on damaged tumor cells. It can be effectively used in the diagnosis and treatment of tumors. By laser-ablating gold materials in water, Torrisi et al. [84] produced spherical gold nanoparticles, transported them to areas with tissue disorders, and decreased their concentration in healthy tissues. With the use of this research, diseased tissues could be easier observed and prepared as a target for radiation and hyperthermia, and tumor cells that protect healthy tissues could be damaged. Stable silver nanoparticles were created by Hashemi et al. [85] using the leaf extract of the medicinal plant T. Polium, and the synthesized nanoparticles significantly inhibited the growth of the MNK45 human gastric cancer cell line, as shown in Figure 14. The study’s data demonstrated the efficacy of Ag NPs as a treatment for poliomyelitis and the potential for further developing anticancer medications. For the treatment of breast cancer cells MCF-7 and MDA-MB-231, Darvish et al. [86] investigated the feasibility of generating Ag NPs from the aqueous extract of leafless flowers. The outcomes demonstrated that the synthesized NPs had an antitumor effect on breast cancer cell lines when applied by upregulating proapoptotic proteins’ expression and downregulating anti-apoptotic proteins’ expression. The biological reduction mechanism based on Ag NPs was contributed by Soltani et al. [87]. The ethanol-water extract of loquat fruit was employed to characterize biosynthetic Ag NPs. Human umbilical vein endothelial cells (HUVECs), which were normal cells, and MCF-7 human breast cancer cells were both shown to have the capacity for apoptosis and anticancer actions.

### 4.3. Others

With the rapid development of nanotechnology, in addition to biomedicine (imaging, sensing, tumor diagnosis and treatment, etc.), precious metal nanoparticles have also been studied, and new attempts have been made in various fields, such as chemical catalysis, toxic detection in food, and sewage detection. The typical application cases of precious metal particles in recent years are summarized in Table 3.

In summary, precious metal NPs are less toxic, biocompatible, and widely used. In recent years, composite materials with doped precious metal particles have been tried in food detection, sewage detection, and waste degradation and achieved excellent results. However, the application of precious metal nanoparticles in clinical or industrial applications requires a large number of experiments and research. In addition, the reunion of metal NPs, their structural strength, large-scale production preparation, and their behavior in a complex biological environment will be the focus of research hotspots in the future.

## 5. Discussions

### 5.1. Similarity

After nearly three decades of development, the laser ablation of Au/Ag NPs has emerged as a convenient and scalable technique for the synthesis of ligand-free nanomaterials in a hermetic environment. Nature has designed various processes for the synthesis of nano as well as microscale organic materials. The biosynthetic method is effective, simple, low-cost, and environmentally friendly. The laser ablation process or biosynthetic method do not need the use of a special environment, such as high temperature and high pressure, but rather require mild conditions and no pollution to the environment, which has attracted more and more attention in many industrial fields [95,96].

In addition to being green manufacturing processes, both synthesis methods can achieve large-scale mass production and reduce the manufacturing cost of NPs. Au/Ag NPs synthesized by laser ablation can be used not only in industrial fields, such as precision optics, catalysts, and energy fields, but also in biological, medical, and other fields. Biosynthetic Au/Ag NPs have very good biocompatibility and can be widely used in biomedical fields. Similarly, due to its relatively low cost, it can also be used in the fields of waste liquid quality and environmental protection [97].

### 5.2. Individuality

Since the processing parameters (e.g., bulk target, solvent and solute, system temperature, and pressure) and laser parameters (e.g., wavelength, pulse energy, number of laser pulses, pulse duration, and repetition frequency) can be flexibly adjusted in the laser ablation method, the laser synthesis process can generate NMS that almost cover the entire periodic table. However, biosynthesis methods are limited by the manufacturing costs of solvents, and the types of NPs they can synthesize are also limited. As shown in Figure 2, the Au/Ag NPs prepared by the laser ablation method are a physical process, which is a top-down method, and the speed to generate the NPs is fast; in contrast, the Au/Ag NPs prepared by biosynthesis represents a bottom-up method, where the formation of the NPs takes a certain amount of time.

Compared with the biosynthesis of Au/Ag NPs, the laser ablation process can also control the process of the synthesized Au/Ag NPs by adjusting the laser parameters, thereby controlling their particle size, shape, production capacity, energy consumption, etc. Due to the many controllable parameters of the laser ablation synthesis of NPs, the size of the prepared NPs ranges from several nm to 8000 nm [23], and their shapes include fractal, fullerene, leaf, hexagonal, flower, football, nanocube, nanowires, nanoneedles, nanoribbons, hollows, core-shells, nanosheets, tubes, nanodisk, etc. However, these shapes are uncommon in biosynthesized Au/Ag NPs.

The particle size distribution of nanomaterials prepared by the physical methods is relatively wide. For example, nanomaterials obtained by the milling method easily agglomerate. Another disadvantage is that these methods often require larger equipment. The nanomaterials prepared by chemical methods have good dispersion, narrow particle size distribution, and uniform morphology, but the surface of the materials may have impurities, which require subsequent treatment.

### 5.3. Complementarity

As discussed above, the laser ablation process and the biosynthetic method both have similarities and differences. How to generate Au/Ag NPs that are high quality, low cost, and produce a large yield has always been a common pursuit of academia and industry. The comparison between the laser ablation process and the biosynthetic method for the fabrication of Au/Ag metal NPs is listed in Table 4. As listed in Table 4, the initial investment cost of the laser ablation process is high, but with the expansion of the mass production scale, the per-gram fabrication cost of its Au/Ag NPs is significantly reduced. In addition, with the continuous development of laser technology, the costs of lasers and their core components will be reduced, and their input costs will also continue to decline. In general, the preparation cost of Au/Ag NPs by biosynthesis is closely related to the selected preparation process, and the initial investment cost is low [39]. However, it is difficult to control the size and shape of Au/Ag NPs via biosynthesis.

Therefore, it is very meaningful to effectively integrate the above two synthesis processes to realize their advantages and avoid their disadvantages. For example, a possible method for the hybrid synthesis of Au/Ag NPs is to pre-prepare Au/Ag NPs by biosynthesis, and then classify them into small, middle, and large categories by a size-screening device. Middle-sized Au/Ag NPs are what we expect and can be output by the size screening device; small-sized Au/Ag NPs continue to undergo biosynthesis and large-sized Au/Ag NPs are fragmented by the subsequent laser ablation process to obtain the desired middle-sized Au/Ag NPs.

## 6. Outlooks

Au/Ag NPs have played a significant role in the fields of biology, medical testing, optical imaging, energy and catalysis, tumor diagnosis and treatment, environmental protection, and so on. For example, they can be used in cell detection, gene regulation for drug synthesis, drug delivery, photo-chemotherapy, and more. The laser ablation and biosynthesis processes are environmentally friendly when synthesizing Au/Ag NPs, meaning they have a negative influence on the environment. As a result, this study focuses on advancements in the laser ablation and biological synthesis of Au/Ag NPs and their applications. In the future, the fundamentals of these two green synthesis processes and their potential applications are anticipated as follows.

(1) Laser synthesis and colloid processing are described as promising synthesis methods with unique properties. However, there is still a lack of effective preparation processes for size control and high productivity, especially with reliable processes that can achieve commercial-scale use. Preparation within some laboratories is also a challenging technical problem when obtaining high productivity. This shows that a preparation cost that is too high will limit the implementation of this process in practical applications. In order to solve this challenge, the core problem is to precisely clarify the LAL mechanism. Whether through experiment or in theory, breaking through its processing mechanism only technically answers the interaction between the laser and substrate, as well as the material ablation process when considering the behavior of bubble dynamics, but it can also be used for more economical means to prepare Au/Ag nanoparticle materials;
(2) The biological method mainly involves plant-mediated synthesis, microbe-mediated synthesis, and algae-mediated synthesis, and the size and shape of the Au/Ag NPs prepared via different biosynthesis methods are different. Few existing studies have addressed the differences between these synthetic methods and how NPs can be prepared using different biosynthetic methods. In the future, the differences between the mechanisms of different biosynthetic methods should be further studied, and understanding how to choose suitable biosynthetic methods to prepare Au/Ag NPs for different application fields also needs attention. In addition, understanding how to rationally combine different biosynthetic methods to prepare Au/Ag NPs of a specific size and shape may also be interesting;
(3) Whether using laser ablation or biosynthesis to create Au/Ag NPs, it is necessary to control their size distribution and shape and the production efficiency/cost of the prepared NPs, which involves process parameter optimization (single-objective or multiobjective optimization). At present, in the field of precision manufacturing, such as that within laser machining, electrical discharge machining, etc., the optimization of the process parameters has been extensively studied, and the optimization algorithm has also been integrated into this equipment. Therefore, for the laser ablation or biosynthesis of Au/Ag NPs, the optimization of the process parameters needs to focus on the synthesis process model. On the one hand, the establishment of these models depends on an in-depth study of their processing mechanisms. The connection between multiple inputs and outputs is established through the “white box” method via a mathematical formula [98]. On the other hand, for the complex synthesis process of Au/Ag NPs, this can also be realized via “black box” methods, such as those represented by regression models [99], neural networks [100,101], etc.

## Figures and Tables

**Table 1:** Comparison of the size and shape of NPs in LAL.

NPs	Methods	Size (nm)	Shape	TEM Images	References
Ag	LAL	100–200 nm	Spherical	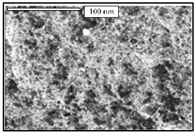	[16]
Ag	LAL	5–140 nm	Spherical	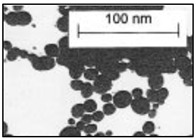	[42]
Ag	LAL.	1.66 ± 0.37 nm	Catenary	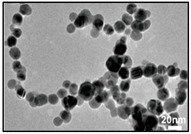	[46]
Au	LAL	20–40 nm	Spherical	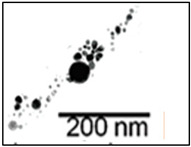	[34]
Au	LAL	20 nm	Spherical	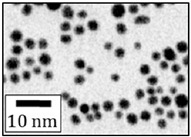	[43]
Au	LAL	20–30 nm	Spherical	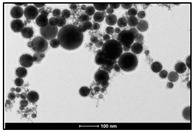	[47]
Au	LAL (nanosecond pulsed)	20 nm	Fractal structures	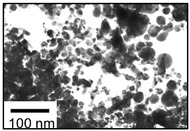	[30]

**Table 2:** Comparison of size and shape in the biological synthesis of Au/Ag NPs.

Methods	NPs	Species	Size (nm)	Shape	References
Plants-mediated synthesis	Au/Ag	Phylloxacin extract	20–100	Spherical-, triangular-, hexagonal- and rod-shaped with irregular contours	[49]
	Ag	*Sterile geranium* leaves extract	20–40	Rods, flat sheets and triangular	[46]
	Au	Pomegranate fruit extract	10–50	Spherical shape	[50]
	Au-Ag	Water leaf extract of wheat	5–30	Spherical to elliptical shapes	[53]
Microbes-mediated synthesis	Au	*V. luteoalbum* and Isolate 6–3	A few to approximately 100	Spherical shape	[56]
	Ag	Fungus “*Fusarium hemitectum*”	10–60	Spherical shape	[57]
	Au	Fungal enzymes	8–40	Spherical and triangular shapes	[59]
	Au	A novel alkalotolerant actinomycete	5–15	Spherical shape	[60]
Algae-mediated synthesis	Au	A novel *Ecklonia cava*	Average particle size of 30 ± 0.25 nm	Spherical and triangular shapes	[68]
	Ag	Australian brown alga *Nostoc algae*	75–2000	Spherical and polydispersed shapes	[71]
	Au	Clover	5–35	Spherical and triangular shapes	[60]
	Ag	Amaranth	5–25	Spherical and triangular shapes	[75]

**Table 3:** Typical application of Au/Ag NPs in recent years grouped by NPs and application fields.

Year	NPs	Application Fields	Remarks	References
2021	Au	Food packaging	Gold nanoparticles can be used in the nano packaging industry.	[88]
2017	Au	Antibacterial nanocomposite material	Gold nanoparticles have non-toxic antibacterial properties against Escherichia coli and Staphylococcus aureus.	[89]
2020	Au/Ag	SERS detection of melamine	The Au/Ag BPHAN array exhibited a strong SERS performance.	[90]
2021	Ag	Sewage detection	The recovery of this method was between 94–97%.	[91]
2021	Ag	Wastewater degradation	The synthesis method of the green nanoparticles can be used for antibacterial and catalytic action.	[92]
2020	Ag	Environmentally friendly cutting fluid	MQL machining assisted by 0.6 wt% AgNP-GCF is 55% more effective in reducing surface roughness.	[93]
2019	Ag	Food packaging	The growth of fungi and bacteria is reduced.	[94]

**Table 4:** Comparison between the laser ablation process and the biosynthetic method for the fabrication Au/Ag metal NPs.

Methods	Advantages	Disadvantages	Remarks
Laser ablation process	Low cost for mass production	The initial investment cost is high, and the process involves many parameters.	An important development direction of mass production in the future
Biosynthetic method	Low initial investment cost	It is difficult to control the size and shape of Au/Ag NP.	The mechanism of the process needs to be further studied to improve the controllability of Au/Ag NPs preparation.
